# Structural Elucidation of Glycosaminoglycans in the Tissue of Flounder and Isolation of Chondroitin Sulfate C

**DOI:** 10.3390/md22050198

**Published:** 2024-04-26

**Authors:** Zhe Wang, Weiwen Wang, Hao Gong, Yudi Jiang, Renjie Liu, Guangli Yu, Guoyun Li, Chao Cai

**Affiliations:** 1 Key Laboratory of Marine Drugs of Ministry of Education, School of Medicine and Pharmacy, Ocean University of China, Qingdao 266003, China; wzhe0427@163.com (Z.W.); wangweiwen97@163.com (W.W.); gonghao0307@163.com (H.G.); 18943348052@163.com (Y.J.); lrjouc@163.com (R.L.); 2 Shandong Provincial Key Laboratory of Glycoscience and Glycotechnology, School of Medicine and Pharmacy, Ocean University of China, Qingdao 266003, China; 3 Laboratory for Marine Drugs and Bioproducts, Qingdao Marine Science and Technology Center, Qingdao 266237, China

**Keywords:** flounder, glycosaminoglycan, HPLC-MS/MS, structure properties

## Abstract

Glycosaminoglycans (GAGs) are valuable bioactive polysaccharides with promising biomedical and pharmaceutical applications. In this study, we analyzed GAGs using HPLC-MS/MS from the bone (B), muscle (M), skin (S), and viscera (V) of *Scophthalmus maximus* (SM), *Paralichthysi* (P), *Limanda ferruginea* (LF), *Cleisthenes herzensteini* (G), *Platichthys bicoloratus* (PB), *Pleuronichthys cornutus* (PC), and *Cleisthenes herzensteini* (CH). Unsaturated disaccharide products were obtained by enzymatic hydrolysis of the GAGs and subjected to compositional analysis of chondroitin sulfate (CS), heparin sulfate (HS), and hyaluronic acid (HA), including the sulfation degree of CS and HS, as well as the content of each GAG. The contents of GAGs in the tissues and the sulfation degree differed significantly among the fish. The bone of *S. maximus* contained more than 12 μg of CS per mg of dry tissue. Although the fish typically contained high levels of CSA (CS-4S), some fish bone tissue exhibited elevated levels of CSC (CS-6S). The HS content was found to range from 10–150 ug/g, primarily distributed in viscera, with a predominant non-sulfated structure (HS-0S). The structure of HA is well-defined without sulfation modification. These analytical results are independent of biological classification. We provide a high-throughput rapid detection method for tissue samples using HPLC-MS/MS to rapidly screen ideal sources of GAG. On this basis, four kinds of CS were prepared and purified from flounder bone, and their molecular weight was determined to be 23–28 kDa by HPGPC-MALLS, and the disaccharide component unit was dominated by CS-6S, which is a potential substitute for CSC derived from shark cartilage.

## 1. Introduction

Glycosaminoglycans (GAGs) are linear polysaccharides composed of repeating disaccharide units and are classified into the following five types: heparin and heparin sulfate (HP and HS), chondroitin sulfate (CS), dermal sulfate (DS), keratin sulfate (KS), and hyaluronic acid (HA) [1,2,3,4]. The biological activities of GAGs are associated with their degree of sulfation, sulfation pattern, and molecular size. Previous studies have reported that the platelet aggregation effect of sulfated DS in plasma is dependent on the content of its substituents [5,6]. The interactions between GAGs and proteins such as interleukin-8 and FGF-2 in cellular regulation processes are affected by the sulfation pattern [7,8]. Therefore, the determination of the type, disaccharide composition, degree of sulfation, and molecular weight of GAGs is important for their structural characterization and biological evaluation.

At present, the conventional sources of natural GAGs are mainly terrestrial animal tissues, such as porcine and bovine cartilage and umbilical cord tissue and chicken crest, while the main marine source is shark cartilage. It is concerning that most of the conventional sources are plagued by problems such as mad cow disease and the ecological crisis. The ocean is an ideal source of GAGs because marine resources are abundant and there is no risk of disease transmission [9]. It has been found that GAGs with unusual acidification patterns are abundant in lower animals such as mollusks and crustaceans (sulfated at the C3 position of GlcN units from the shrimp *Litopenaeus vannamei* and sulfated at the C4 and C6 positions of GalNAc from diamond squid *Thysanoteuthis rhombus*) [10,11]. In addition, CS from sea cucumber is fucosylated chondroitin sulfate and unique with promising anticoagulant activity [12,13]. The structural analysis of GAGs from marine fish has previously been concentrated on tissues such as skin, cartilage, and vertebrae from sturgeon or shark (sulfated at the C4 or C6 position of GalNAc) [14,15,16,17,18]; on the contrary, research on GAGs in muscle tissue is rare.

The detailed structure of GAGs is highly related to their biological activity, so it is of great significance to obtain the structure information of GAGs. The traditional strategy to elucidate GAG structures involves a combination of techniques such as FT-IR, agarose gel electrophoresis, NMR, and molecular weight determination after a tedious separation process [19,20,21,22,23,24]. Therefore, the HPLC-MS/MS technique is a good choice to characterize polysaccharides more quickly and accurately, combing the analysis of unsaturated disaccharide products with the enzymatic digestion, which is thought to be a powerful method for confirming and quantifying the structural properties of GAGs from different biological sources.

Here, the benthic teleost flatfish was selected as the research object and analyzed by HPLC-MS/MS; 16 disaccharide structures were identified, and the contents of HS, CS, and HA were quantified. Our study on the detailed information of the structure and content of GAGs in flatfish tissues provides a rapid and high-throughput method for bioresource screening. Through the guidance of HPLC-MS/MS analysis, we prepared and purified four kinds of CS from flounder bones and determined their molecular weight and structural composition in detail.

## 2. Results and Discussion

### 2.1. Data Processing

**Figure 1:**
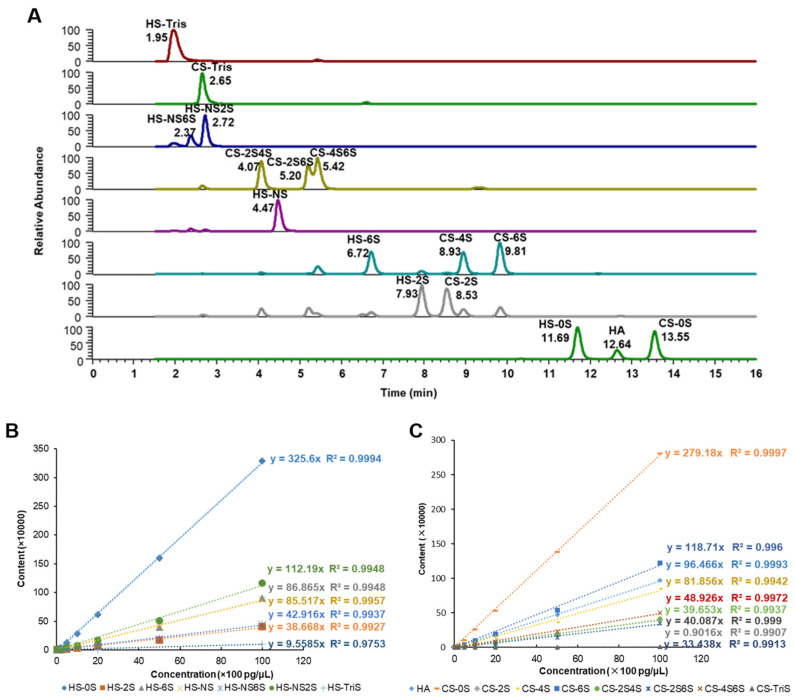
Calculation of disaccharide samples digested from GAGs. (**A**) MRM chromatograms of 2-AMAC-labeled disaccharides. Calibration curves of 2-AMAC-labeled (**B**) HS, (**C**) CS and HA disaccharide standards.

MRM is a highly sensitive analysis mode in LC-MS/MS, which is very suitable for the detection of GAGs in tissue samples with a complex composition [25,26,27,28]. Here, the MRM of the 2-AMAC-labeled seven HS-derived disaccharides, eight CS-derived disaccharides, and one HA-derived disaccharide were isolated (Figure 1A). The linear equation describing the correlation between the relative abundance of each disaccharide in the range from 20 pg to 10,000 pg and the concentration is given in Table S1. The standard curves (Figure 1B,C) were used to calculate the content of each derived standard disaccharide in the sample. After calculation, the percentage of each disaccharide in different GAGs was listed.

### 2.2. Content and Disaccharide Composition of CS in Different Flounder Tissues

**Figure 2:**
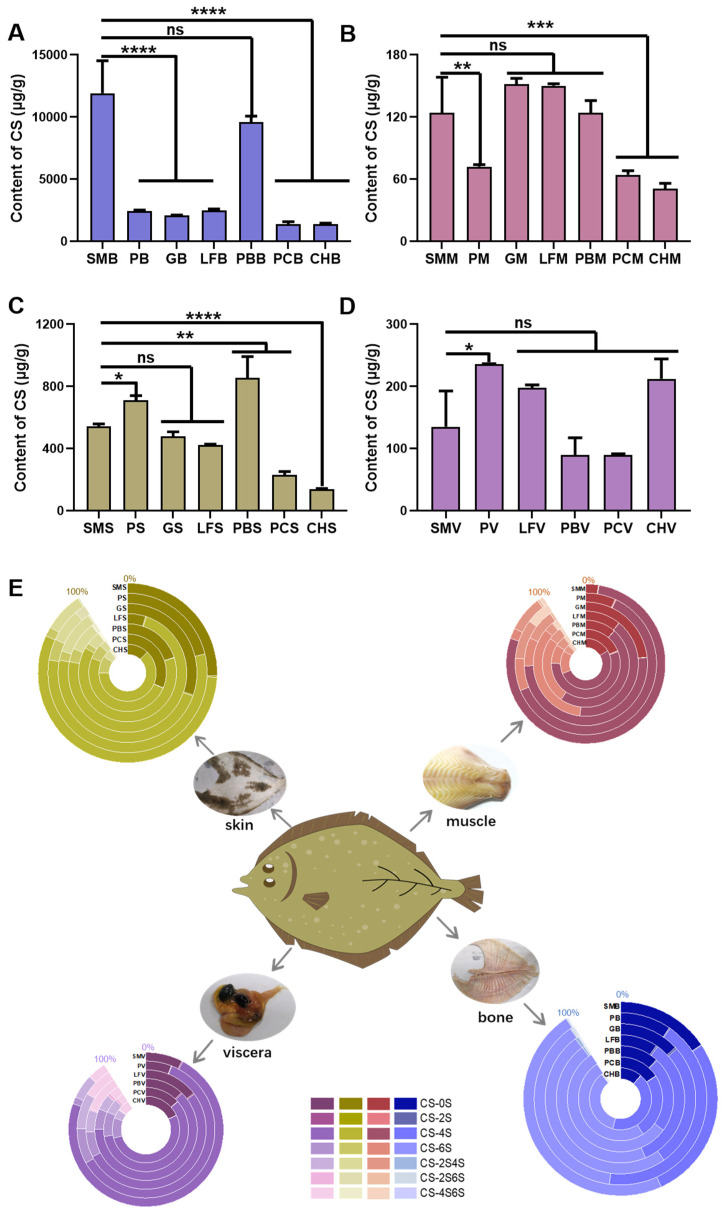
(**A**–**D**) The content of CS in (**A**) bone, (**B**) muscle, (**C**) skin, and (**D**) viscera of seven flounders. (**E**) CS disaccharide composition. Data are shown as the mean ± SD (*n* = 3) with error bars representing standard errors. * *p* < 0.05, ** *p* < 0.01, *** *p* < 0.001, **** *p* < 0.0001, ns *p* > 0.05.

CS, HS, and HA isolated from fish and invertebrates have been investigated only in a limited number of species. Here, we performed content and composition analysis on different kinds of flounder tissue. We analyzed seven different species of flounder, namely *S. maximus* (SM), *Paralichthys* (P), *L. ferruginea* (LF), *C. herzensteini* (G), *P. bicoloratus* (PB), *P. cornutus* (PC), and *C. herzensteini* (CH). Among them, *L. ferruginea* and Canadian Gray Plaice are from the Canadian deep sea, and other flounders are from the Bohai Sea and the Yellow Sea, near China. Bone, muscle, skin, and viscera dissected from each flounder (Canadian Gray Plaice has no viscera) were obtained for GAG analysis. GAG contents are expressed as nanograms per milligram of dry tissue. CS is a sulfated GAG composed of a disaccharide unit composed of *N*-acetyl galactosamine (GalNAc) and glucuronic acid (GlcA) linked by alternative β-1,4 bonds and β-1,3 bonds. Based on the various sulfation sites and levels, it can be categorized into CSA, CSC, CSD, and CSE. CSA predominantly undergoes sulfation at *O*-4 of GalNAc, while CSC occurs at *O*-6 of GalNAc. In addition, CSD is distinguished by the sulfation at *O*-6 of GalNAc and *O*-2 of GlcA, whereas CSE involves two sulfations, at both the *O*-4 and *O*-6 positions of GalNAc. At present, marine CS is mainly extracted from fish cartilage, and there is a problem of resource scarcity [29]. CS content varied significantly between bone, muscle, skin, and viscera of flounder (Figure 2A–D). Obviously, CS content in fish bone is notably higher than that in other tissues, ranging from 1 to 13 μg/mg. This finding is consistent with the published data on the level of uronic acid in sturgeon tissues [30]. In comparison, CS content in other tissues is less than 1 μg/mg; therefore, bone of fish is an attractive source of CS. The concentration of CS in the bone of *S. maximus* and *P. bicoloratus* was found to be the highest, with no significant difference between them. However, there was a notable decrease in CS levels in the bone of the other five flounder species. As an inedible part, abundant fish bones are used to extract CS, which is a reasonable use of resources [30]. In addition, the CS content and structure in the same tissue derived from different species of fish also showed a difference. For example, the CS contents in fish bones are shown in Figure 2A and Table S2. It is clearly indicated that the CS content of SMB is the highest, up to 12 μg/mg in dry samples, followed by SBB. The CS content in bone of deep-sea flounders and *Paralichthys* is about 2 μg/mg, and the lowest CS content in bone is observed for PCB and CHB, only at around 1 μg/mg. There are significant differences in CS content among different species, and their potential as GAG resource is also varying. Rapid analysis of CS content in tissues by HPLC-MS/MS is a fast and convenient method for searching excellent biological resources.

The biological activity of CS is closely associated with their structural properties. The detailed structure of CS in flounder tissues was subsequently analyzed with the above LC-MS/MS data. The abundances of CSA and CSC were the highest in each tissue, followed by unsulfated CS and a low percentage of disulfide CS (Figure 2E and Table S3). A consistent feature of muscle, skin, and viscera tissues was that the percentage of CSA was higher than that of CSC, and the CS composition is similar in skins of gray triggerfish, smooth hound, Sciaena umbra, and our flounders [17,31]. Interestingly, a different characteristic was found in the fish bones. The percentage of CS 4S (46.56 and 46.70%, respectively) was higher than that of CS 6S (41.82 and 39.17%, respectively) in YPB and PKB, but CS 6S was dominant in the other five flounders (SMB 52.16%, GB 54.50%, LFB 50.83%, PBB 61.20%, GYB 54.57%). It was found that the dominant CS 6S content was not unique to deep-sea flounders, and the distinct species were important reason for this content difference. Similar to our research results, CS 6S was the main CS structure in the fish bones of Yellowfin sole (a bottom and deep-sea flatfish) [32]. The CS structure of fish bones could not be determined in the living environment of fish, as there is no reference or theoretical basis. Therefore, the HPLC-MS/MS high-throughput screening method used in this study is a reliable means to find the ideal source of CS.

### 2.3. Content and Disaccharide Composition of HS in Different Flounder Tissues

**Figure 3:**
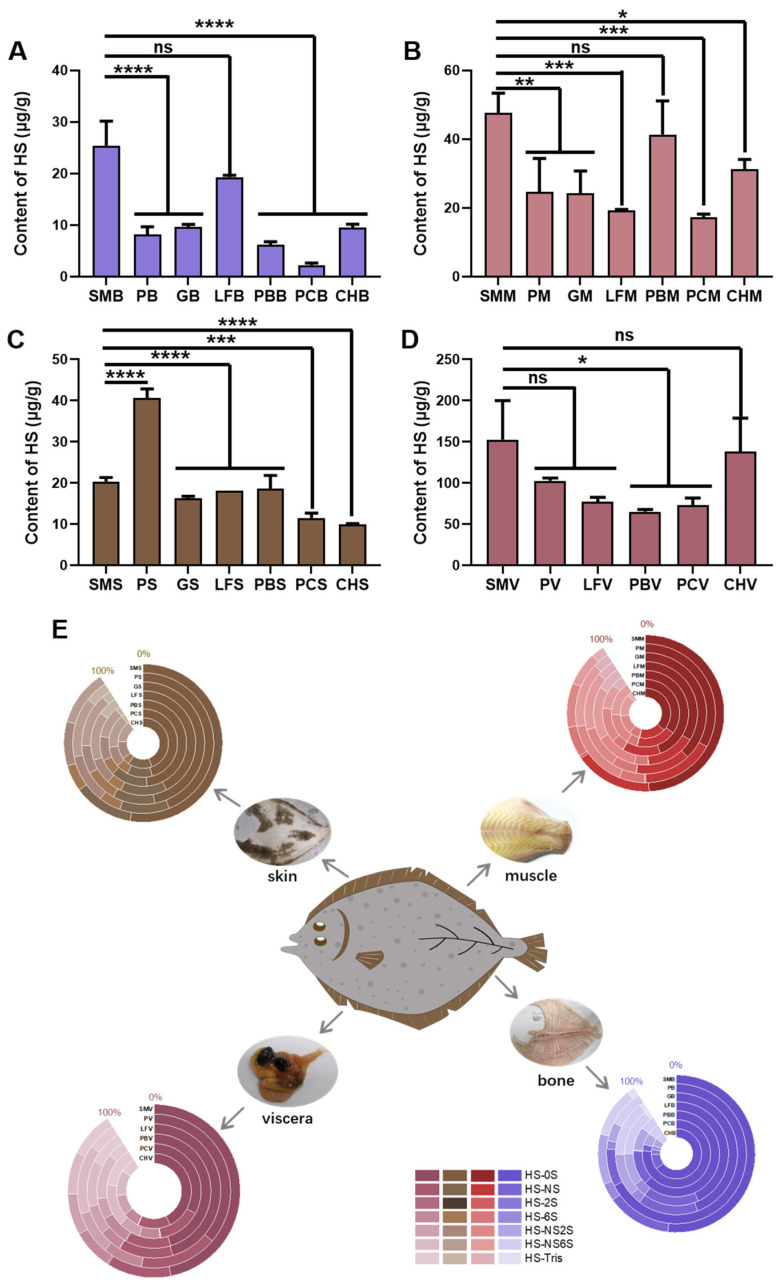
(**A**–**D**) The content of HS in (**A**) bone, (**B**) muscle, (**C**) skin, and (**D**) viscera of seven flounders. (**E**) HS disaccharide composition. Data are shown as the mean ± SD (*n* = 3) with error bars representing standard errors. * *p* < 0.05, ** *p* < 0.01, *** *p* < 0.001, **** *p* < 0.0001, ns *p* > 0.05.

HS is a class of polyanionic polysaccharides composed of *N*-acetyl glucosamine (GlcNAc) and glucuronic acid (GlcA)/iduronic acid (IdoA) repeating units linked by β-1,4 bonds with variable sulfation patterns. The biological source of heparin as an anticoagulant drug and HS as a byproduct of the heparin manufacturing process are mainly from the small intestine of mammals, while their marine source is mollusks. There are very few studies on HS in flounder. The content of HS in flounder bone, muscle, skin, and viscera is significantly lower than that of CS (Figure 3A–D and Table S2) reported in our study. *S. maximus* exhibits the highest HS content in bone, muscle, and viscera. The HS content of *L. ferruginea* in bone is similar to that of *S. maximus* with no significant difference, while other flounder species show significantly lower content. In muscle, the HS content of PB closely resembles that of *S. maximus*. There is no significant difference in HS content among species in viscera. Significant differences in HS are observed among species in fish skin, with PS content being the highest. The amount of HS was highly variable, ranging from 70 to 160 ng/mg in viscera with the highest value at 152 ng/mg in SMV and from 2 to 50 ng/mg in other tissues with the lowest content at 2.25 ng/mg in PCB. Of note, disaccharides of HS are abundant. HS disaccharide analysis indicated that unsulfated disaccharide (HS-0S), monosulfated disaccharide (HS-NS, HS-2S, HS-6S), disulfated disaccharide (HS-NS2S, HS-NS6S), and trisulfated disaccharide (HS-NS2S6S, HS-TriS) were all clearly detected in flatfish tissues. It is indicated that HS-0S had the highest relative abundance in all tissues, accounting for 36–75%, whereas HS-TriS had the lowest relative abundance, accounting for less than 15%. The relative abundance of other types of HS disaccharide varied slightly between different species and tissues; specific data are shown in Figure 3E and Table S4. The low amount in tissues makes it very challenging to separate and purify HS-related polysaccharide. Nogueira et al. used anion exchange chromatography to extract and purify GAG from total fish viscera of *Oreochromis niloticus* and *Piaractus mesopotamicus* without obtaining purified HS even after double fractionation [33]. Compared with mammals, the extraction of HS from flounders poses a great challenge due to the low content of HS in flatfish with lower sulfation degree as well. Thus, HPLC-MS/MS high-throughput screening is a promising approach to characterize the HS structures in detail.

### 2.4. Content of HA in Different Flounder Tissues

**Figure 4:**
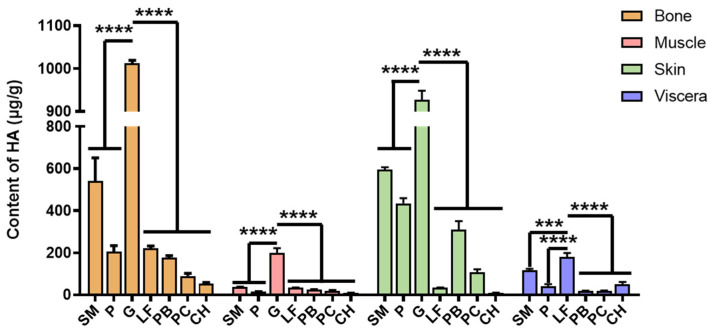
The HA content in bone, muscle, skin, and viscera of seven flounders. Data are shown as the mean ± SD (n = 3) with error bars representing standard errors. *** *p* < 0.001, **** *p* < 0.0001.

HA is a unsulfated GAG composed of a disaccharide unit composed of *N*-acetyl glucosamine (GlcNAc) and glucuronic acid (GlcA) linked by β-1,3 bonds derived from animal skin, synovia, and vitreous. In this study, we found that the amount of HA was relatively high in bone (Figure 4 and Table S2); for example, it was higher than 1 μg/mg in GB. This may be attributed to the higher levels of joint synovial fluid in the bones. On the contrary, the HA content was relatively low in muscle; for example, it was only 8.13 ng/mg in CHM. In most flounder species, the HA content is 100–600 ng/mg. Of note, in the present study, the HA content in skin was the highest (927.24 ng/mg) in GS, but it was only 34.53 and 10.51 ng/mg in LFS and CHS, respectively. In contrast, the HA content in LFV was 178.82 ng/mg, which was higher than that of other viscera samples (18–150 ng/mg). On the whole, the HA content of Canadian Gray Plaice was the highest among the same tissues (viscera were not included in the comparison) and exhibits significant differences in bone, muscle and skin compared with other species. In terms of viscera, *L. ferruginea* has the highest content of HA, which is also significantly higher than that of other species. In *L. ferruginea*, which also lives in the deep sea, the HA content was relatively lower, except in viscera.

### 2.5. The Sulfation Degree of CS and HS in Flounder Tissue

The degree of sulfation is a crucial indicator to describe the sulfation levels of series of GAG. Some authors have proposed different biological roles of GAG, such as the signaling function of various growth factors and anticoagulant function, which are closely related to the sulfation pattern of GAG. In addition, CS sulfation mode has an effect on cartilage formation in mesenchymal stromal cells (MSCs), and CS-4 and -6 isoforms can promote cell proliferation and may promote cartilage [34]. It is presented by calculating the number of sulfonation in the monosaccharides. Because HA is a non-sulfated GAG, the sulfation degree is 0. Furthermore, the sulfation degree of CS and HS in flounder tissue is shown in Table 1. The degree of CS sulfation was 0.64–1.05, whereas the degree of HS sulfation (0.23–1.03) was lower. CS and HS are relatively abundant in sulfated disaccharide in muscle and viscera, so their degree of sulfation is higher than that of other tissues. In general, this may be related to the importance and diversity of their functions in organisms.

### 2.6. Structural Analysis of CS from Fish Bone

According to the above HPLC-MS/MS results, flounder bones are abundant in CS with a high ratio of CS-6S. Based on this, CSC, namely SMBCS, PBBCS, LFBCS, and GBCS, was extracted and purified from bone tissue of *S. maximus*, *P. bicoloratus*, *L. ferruginea*, and Gray Plaice. The total amount of CS was analyzed after elution by a QFF column. SMBCS was used as a reference, which showed that the highest CS content was recovered in 0.8 M sodium chloride eluate (Figure S1), so this condition was used for all other samples’ processing.

**Figure 5:**
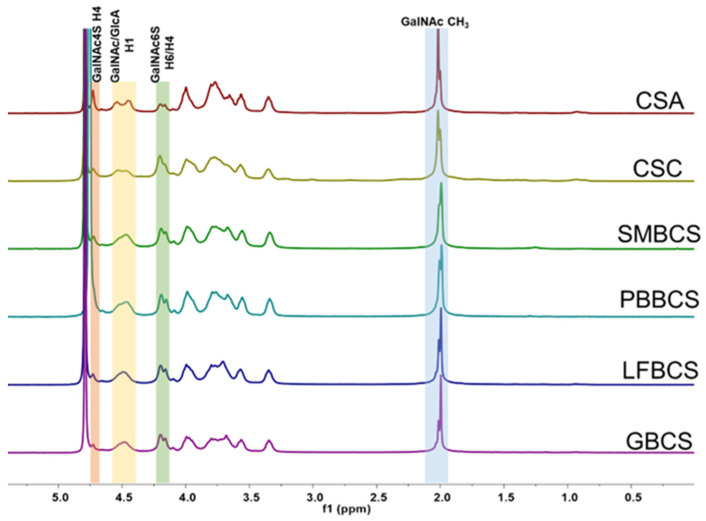
^1^H-NMR spectra of CS from different sources.

The biological activity of polysaccharides is dramatically affected by the molecular weight distribution. The optimized molecular weight of polysaccharides can be achieved by reducing the apparent viscosity, increasing the solubility of water, and promoting the passage through the intestinal mucosa and gastric mucosa, etc. Low molecular weight CS from bovine nasal cartilage has been reported to have good antioxidant activity, with a molecular weight of 19.7 kDa and a scavenging rate at 36% of the •OH radical [35]. The molecular weight (Mw) of different sources of CS as determined by HPGPC-MALLS is shown in Figure 5. The signal peak shape is symmetrical and clear, indicating that the purity of the sample is high with a narrow distribution range. The Mw of purchased and extracted CS is summarized in Table S5. The largest Mw was observed in CSE derived from squid cartilage (146.40 kDa) and CSC derived from shark cartilage (56.07 kDa), which is consistent with existing reports (50–60 kDa) [21]. The Mw of SMBCS, PBBCS, LFB, and GB was 25.40, 23.98, 27.76, and 24.08 kDa, respectively (Table S5 and Figure S2), which were all lower than that of CS derived from cartilaginous fish (>40 kDa) [36]. Of note, the samples from *S. maximus*, *P. bicoloratus*, *L. ferruginea*, and Gray Plaice showed very similar properties, making a variety of flounder an adequate source of CS with similar Mw. Among them, CSA derived from bovine trachea tissue has two more obvious peaks; the rest of the samples are dominated by single peaks. The peak before 20 min in the HPLC data of SMBCS can be ignored, because in the original data (Figure S2), the peak did not show a differential refractive index; this artifact may be caused by the detection of large particulate matter.

The signal in ^1^H-NMR is clearly concentrated in two spectral regions (Figure 5). The peaks between 1.9 ppm and 2.1 ppm are exactly assigned to be the hydrogen signal of acetyl groups, including two characteristic signals from GalNAc 4S (2.01 ppm) and GalNAc 6S (1.99 ppm), respectively. Meanwhile, the peaks between 3 ppm and 5 ppm are the proton signal on sugar rings as shown. Signals at 0.5–1.5 ppm and 7.0–8.5 ppm are absent, which clearly confirms that it does not contain aliphatic and aromatic amino acids as well. Furthermore, no characteristic peaks of dermatan sulfate are observed in the spectrum (H1 is 4.87 ppm, H2 is 3.52 ppm of the iduronic acid residue), indicating high CS purity.

**Figure 6:**
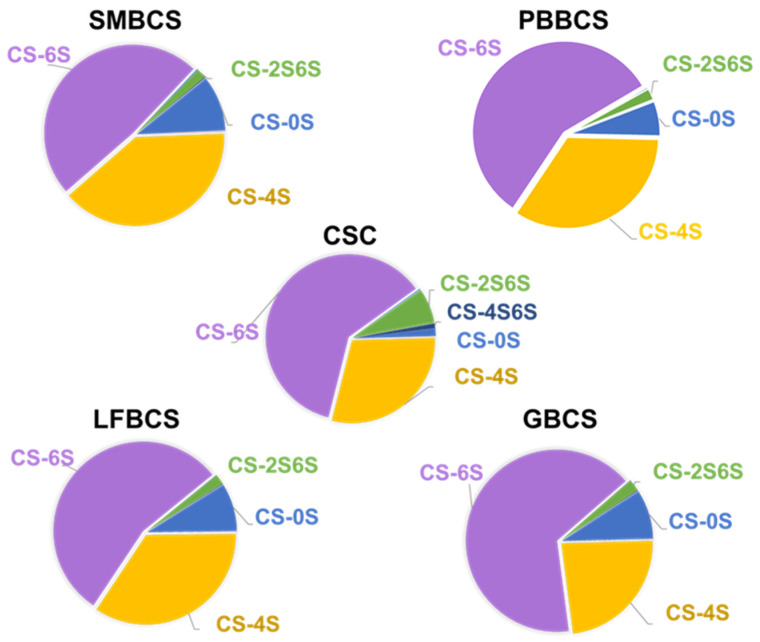
Disaccharide composition of CS from different sources.

Combining the two-dimensional spectra (Figures S3 and S4) with existing reports [20,21,36], the ^1^H-NMR and ^13^C-NMR signals for CSA and LFBCS are attributed. In ^1^H-NMR, the anomeric hydrogen H1 signals of GalNAc and GlcA are assigned at 4.53 and 4.46 ppm, respectively. The H2 and H3 signals of GlcA were found at 3.35 and 3.57 ppm, respectively. In addition, the H2 signal of GlcA2S was observed at 4.09 ppm. The H6 signals of GalNAc6S and GalNAc4S are distributed at 4.20 and 3.80 ppm, respectively. The H4 signals of GalNAc6S and GalNAc4S were clearly observed at 4.16 and 4.73 ppm, and the 4S/6S ratios were calculated to be 2.32 (CSA) and 0.76 (LFBCS) based on their peak area. In the ^13^C-NMR spectra, compared with GalNAc4S, the intensity of the characteristic carbon signal corresponding to GalNAc6S of LFBCS is dominant, and it is determined that CS-6S is dominant in LFB, while in CSA, on the contrary, CS-4S is dominant. Other hydrogen signals are clustered at 3.90–4.05 ppm and 3.60–3.83 ppm, making them difficult to distinguish. The chemical shift signals in the carbon spectrum are annotated in Figures S3B and S4B. NMR spectra of CS from different sources are shown in Figure 6. The signal peak shapes in the ^1^H-NMR spectra of LFBCS, GBCS, SMBCS, PBBCS, and CSC are very similar, indicating that those CSs are composed of similar disaccharide components. The H4 characteristic signal intensity of GalNAc6S and GalNAc4S can be used to determine the main disaccharide composition of CS, and here, it is certain that all CS extracted from fish bone is CSC (CS-6S is the main disaccharide component).

The analysis of unsaturated disaccharide composition of SMBCS, PBBCS, LFBCS, GBCS, and CSC is shown in Figure 6 and Table S6. It can be seen that the five CSs are dominated by CS-6S, among which the proportion of CS-6S in GB is the highest (65.67%), followed by commercial CSC (61.12%), while CS-6S accounts for 57.48, 54.78, and 48.47% in PBBCS, LFBCS, and SMBCS, respectively. The 4S/6S ratios for CS from different sources are 0.81 (SMBCS), 0.60 (PBBCS), 0.63 (LFBCS), and 0.36 (GBCS). Compared to the tissue analysis in Section 2.2 and Section 2.3 (Figure 3 and Table S3), the proportion of CS-6S in GB and LFB was increased, while the proportion was decreased in SMB. The reason for this difference is caused by the purification process. PBB, GB, and LFB effectively separated the components of CS-0S under low salt concentration conditions, resulting in a decrease in the proportion of fragments with CS-0S and strengthening the purification of fragments with CS-6S, while SMB was eluted. In addition to the separation of low-sulfated fragments, the CS-6S containing part was also separated, resulting in a close proportion of fragments with CS-4S and CS-6S. Overall, CS from flounder bone is very suitable as a substitute for shark-derived CSC. It is worth mentioning that its raw materials are easy to obtain, the obtained CS structure is mainly CS-6S, the molecular weight is uniform, and the purification process is relatively easy.

## 3. Materials and Methods

### 3.1. Materials and Chemicals

*Limanda ferruginea* (LF) and *Cleisthenes herzensteini* (Canadian Gray Plaice; G) derived from the Canadian deep sea were purchased from OCEAN DIARY (Qingdao, China). *Scophthalmus maximus* (SM), *Paralichthys* (P), *Platichthys bicoloratus* (PB), *Pleuronichthys cornutus* (PC), and *Cleisthenes herzensteini* (CH) derived from the coastal waters of Qingdao were purchased from the Tuandao aquatic products market of Qingdao (Shandong, China). All flounder tissues were stored at −20 °C immediately after dissection.

The reversed-phase Luna C18 chromatography column (50 × 2.1 mm, 3 μm) was obtained from Phenomenex (Torrance, CA, USA). Pronase was purchased from Roche (Basel, Switzerland). 2-Aminoacridone (2-AMAC), NaBH_3_CN, methyl alcohol, and ammonium acetate were purchased from Sigma-Aldrich (St. Louis, MO, USA). GAG-lyases (heparin lyases I, II, and III and chondroitinases ABC, AC, and B) were obtained from Beijing Bicheng Biotechnology Co., Ltd. (Beijing, China). All other chemicals were obtained from Sigma-Aldrich and were of analytical grade.

The unsaturated HA disaccharide ΔUA-GlcNAc/HA 0S, the unsaturated HS disaccharide standards ΔUA-GlcNAc/HS 0S, ΔUA-GlcNS/HS NS, ΔUA-GlcNAc6S/HS 6S, ΔUA2S-GlcNAc/HS 2S, ΔUA2S-GlcNS/HS NS2S, ΔUA-GlcNS6S/HS NS6S, and ΔUA2S-GlcNS6S/HS TriS, and the unsaturated CS disaccharide standards ΔUA-GalNAc/CS 0S, ΔUA2S-GalNAc/CS 2S, ΔUA-GalNAc6S/CS 6S, ΔUA-GalNAc4S/CS 4S, ΔUA2S-GalNAc4S/SB, ΔUA2S-GalNAc6S/SD, ΔUA-GalNAc4S6S/SE, and ΔUA2S-GalNAc4S6S/CS TriS were purchased from Iduron (Manchester, UK).

### 3.2. Sample Preparation and Labeling

The seven kinds of flounder were dissected manually to obtain bone, muscle, skin, and viscera tissues. After drying at 70 °C, the samples were crushed with an electric grinder to produce a homogeneous powder. To ensure the complete degreasing of every sample, they were ultrasonicated (1 mg per sample) in acetone for 20 min and then centrifuged at 7800× *g* at 25 °C for 10 min. The precipitate was dried in a fume cupboard. The dried precipitate was subjected to digestion at 50 °C for 12 h with 2.5% pronase (5 mg/mL). Proteases were inactivated by heating at 100 °C for 10 min. The supernatant was collected by centrifugation (13,000× *g*, 15 min) and 4 volumes of 95% ethanol were added. After precipitation at 4 °C for 12 h and further centrifugation (13,000× *g*, 10 min), the precipitate was collected and further dried in a fume cupboard.

Next, each sample was dissolved in 300 μL of digestion buffer (20 mM ammonium acetate, 1 mM calcium chloride, pH 7.0) and degraded in a cocktail of GAG lyases (30 mU/enzyme) for 8 h at 37 °C. After inactivation, the resulting disaccharides were recovered by centrifugation, removing the precipitate, and freeze-dried.

Finally, the recovered disaccharide samples together with unsaturated disaccharides standards were labeled by reductive amination with 2-AMAC. The specific steps were as follows. Disaccharide samples were labeled using 10 μL of 0.2 M 2-AMAC solution in glacial acetic acid–DMSO (3:17, *v*/*v*) and incubated at room temperature for 30 min. Next, 10 μL of a freshly prepared solution of 2 M sodium cyanoborohydride in water was added. Then, derivatization was carried out by incubation at 45 °C for 4.5 h in the dark. Finally, the reaction mixture was diluted five times by the initial mobile phase and centrifuged to prepare the supernatant for LC-MS/MS analysis.

### 3.3. HPLC-MS/MS Analysis

LC-MS/MS was carried out on a triple quadrupole mass spectrometer (Thermo Fischer Scientific, Waltham, MA, USA) in multiple reaction monitoring (MRM) mode. The separation was performed with a Thermo UltiMate 3000 HPLC separation system on a Luna C18 column (2 × 100 mm, 3 μm, Torrance, CA, USA) at 45 °C. Eluent was passed through the column at a flow rate of 120 μL/min with the following gradient: 0–2 min, 10% B; 2–12 min, 10–25% B; 12–17 min, 25% B. Quantitative analysis of 2-AMAC-derived disaccharides was performed by using 2-AMAC-derived disaccharide standards (20, 40, 200, 1000, 5000, and 10,000 pg per disaccharide) to construct calibration curves. In MRM mode, the linearity was evaluated according to the amount and peak intensity of disaccharides. All analyses were performed in triplicate.

### 3.4. Extraction and Purification of CS from Flounder Bone

The bones obtained by dissection of *S. maximus*, *P. bicoloratus*, *L. ferruginea*, and Canadian Gray Plaice were dried and crushed. SMBCS, PBBCS, LFBCS, and GBCS were extracted and purified according to a previously described method [37]. Briefly, 10 g of fishbone powder was degreased by soaking in 30 mL of methanol/chloroform (3:1, *v*/*v*) for 24 h. The pellet was incubated overnight in 100 mL 1% NaOH solution, after which the pH was adjusted to 7 with 3 M hydrochloric acid. Next, 100 mL of 0.2 M sodium acetate buffer solution (pH 6.0), 1 g papain, 10 mM EDTA-2Na, and 10 mM cysteine were added to the solution, and the mixture was stirred at 60 °C for 24 h, followed by inactivation at 100 °C for 10 min. The samples were centrifuged and the supernatant was retained. Next, 800 mL of ethanol was added to the supernatant, followed by incubation overnight at 4 °C. After centrifugation at 8000× *g* for 10 min, the precipitate was obtained. This precipitate was dissolved in distilled water, and crude polysaccharides were obtained after dialysis and freeze-drying.

The obtained crude polysaccharides were separated by Q-Sepharose Fast Flow strong anion exchange columns (3 × 30 cm) and eluted with 0.4, 0.6, 0.8, 1.0, and 2.0 M NaCl solutions, and the components were collected for dialysis and freeze-drying. The samples were then redissolved with 16% NaCl solution, four volumes of absolute ethanol were added to precipitate the CS, and the above operation was repeated three times to complete the CS purification.

### 3.5. Molecular Weight Determination by HPGPC-MALLS

The molecular weights of SMBCS, PBBCS, LFBCS, and GBCS were elucidated by an Agilent 1260 HPLC platform (Agilent, Santa Clara, CA, USA) equipped with a Dawn Heleos-II multiangle laser light scattering instrument and refractive index detector (Wyatt Technology, Santa Barbara, CA, USA). Gel chromatography was performed with Shodex OHpak SB-804 HQ and SB-802.5 HQ columns at a flow rate of 0.6 mL/min and with a mobile phase of 0.1 M Na_2_SO_4_. The sample concentration was 5 mg/mL, and the sample was analyzed at 30 °C for 45 min.

### 3.6. Structural Analysis of CS by NMR and HPLC-MS/MS

Pure polysaccharide (10 mg) was dissolved with 200 μL D_2_O and concentrated at low pressure until it was completely dry. This procedure was repeated three times, and then the sample was transferred to a nuclear magnetic tube and dissolved in 600 μL D_2_O. An Agilent DD2-500 MHz nuclear magnetic resonance spectrometer (Agilent Technologies, Santa Clara, CA, USA) was used for nuclear magnetic resonance spectroscopy analysis. One-dimensional 1H-NMR and ^13^C-NMR and two-dimensional ^I^H-^1^H COSY and ^1^H-^I3^C HSQC analyses were conducted at 298 K.

Polysaccharide (1 μL per sample, concentration 10 μg/μL) was diluted with 200 μL digestion buffer, and then chondroitinase (10 mU each for ChABC, ChAC, and ChB) was added to break it into disaccharides. The subsequent labeling and HPLC-MS/MS procedures were the same as those described in Section 3.2 and Section 3.3. The CS content of the components eluted by QFF was determined and the composition of the purified CS was analyzed.

## 4. Conclusions

In this study, the differences of GAGs fraction from different tissues of flounders were elucidated by systematical analysis of their structural properties. It was found that flounder bone was the first choice for the preparation of CS 4S/6S. The bones of *S. maximus*, *P. bicoloratus*, *L. ferruginea*, and *C. herzensteini* represent novel marine sources of chondroitin sulfate (CSC), with a structure analogous to shark-derived CSC. The heparan sulfate (HS) content in all flounder tissues is insufficient for potential production, ranging from 10–150 μg/g, primarily concentrated in the viscera of *S. maximus*; its structure was found to be HS-0S without sulfation, followed by HS-NS with sulfated amino groups. Hyaluronic acid (HA) without sulfation modification is predominantly present in the skin and to a lesser extent in the bones of the flounder. Deep-sea *C. herzensteini* exhibited superiority with a HA content of 1012 μg/g and 927 μg/g in fish bone and skin, respectively, indicating its potential for development. We provided a high-throughput rapid detection method for tissue samples by using HPLC-MS/MS to rapidly screen ideal sources of GAGs. Through the guidance of screening results, CS with a uniform molecular weight and a high content of CS-6S was successfully prepared and purified from the bone tissue of four flounder species, which can be used as a substitute for shark cartilage-derived CSC.

## Figures and Tables

**Table 1:** The degree of CS and HS sulfation in flounder tissue.

Sample		SM	P	LF	G	PB	PC	CH
CS-S	bone	0.80	0.88	0.89	0.84	0.85	0.80	0.87
	muscle	1.05	1.05	0.90	0.78	0.91	0.82	0.86
	skin	0.74	0.81	1.02	0.71	0.79	0.64	0.90
	viscera	0.98	1.07	0.96	-	0.93	0.91	0.86
HS-S	bone	0.60	0.34	0.73	0.72	0.40	0.23	0.34
	muscle	0.60	0.95	0.99	0.86	0.74	0.87	0.85
	skin	0.56	0.78	0.79	0.67	0.63	0.71	0.71
	viscera	0.71	0.87	0.75	-	0.71	1.03	0.77

## Data Availability

The data presented in this study are available on request from the corresponding authors.

## Supplementary Materials

The following supporting information can be downloaded at: https://www.mdpi.com/article/10.3390/md22050198/s1, Table S1: Linear equation of GAGs disaccharide standard curve; Table S2: Average quantity of GAG derived from flounders; Table S3: Percentage of CS disaccharide compositions Table S4. Percentage of HS disaccharide compositions; Table S5. Molecular weight of CS from different sources; Table S6. Percentage of CS from the flounder bone disaccharide compositions; Figure S1: Analysis of CS composition and content of polysaccharide eluting components of SMB; Figure S2: HPGPC-MALLS chromatograms of (A) CSE, (B) CSA, (C) CSC, (D) SMBCS, (E) PBBCS, (F) LFBCS and (G) GBCS; Figure S3: (A) ^1^H-NMR, (B) ^13^C-NMR, (C) ^1^H-^1^H COSY and (D) ^1^H-^13^C HSQC spectroscopy of CSA; Figure S4: (A) ^1^H-NMR, (B) ^13^C-NMR, (C) ^1^H-^1^H COSY and (D) ^1^H-^13^C HSQC spectroscopy of LFBCS.
