# Supplementary material for: Structural Elucidation of Glycosaminoglycans in the Tissue of Flounder and Isolation of Chondroitin Sulfate C
Source: Mar Drugs. 2024 Apr 26;22(5):198. doi: 10.3390/md22050198 (PMC11123320; doi:10.3390/md22050198)
Supplement: Supplementary file 1 [file marinedrugs-22-00198-s001.zip › marinedrugs-2965989-supplementary.pdf]

Structural elucidation of glycosaminoglycans in the tissue of flounder and isolation of chondroitin sulfate C

Table S1. Linear equation of GAGs disaccharide standard curve.

Disaccharide standard	Linear equation	R2
HS-TriS	y = 9.5585x	0.9753
CS-TriS	y = 0.9016x	0.9907
HS-NS6S	y = 42.926x	0.9937
HS-NS2S	y = 112.19x	0.9948
SB (CS-2S4S)	y = 39.653x	0.9937
SD (CS-2S6S)	y = 33.43x	0.9913
SE (CS-4S6S)	y = 48.926x	0.9972
HS-NS	y = 85.517x	0.9957
HS-6S	y = 86.865x	0.9948
CS-4S	y = 81.856x	0.9942
CS-6S	y =118.71x	0.9960
HS-2S	y = 38.668x	0.9927
CS-2S	y = 40.087x	0.9990
HS-0S	y = 325.6x	0.9994
HA-0S	y =96.466x	0.9993
CS-0S	y = 279.18x	0.9997

Table S2. Average quantity of GAG derived from flounders.

		Bone	Muscle	Skin	Viscera	total amount
average quantity of CS (μg/g)	SM	11917.76	146.88	543.88	260.05	12868.57
	P	2435.40	72.04	711.91	236.45	3455.80
	G	2101.92	151.87	478.97	-	2732.77
	LF	2470.63	150.20	423.77	198.15	3242.75
	PB	9607.09	124.17	854.51	89.80	10675.57
	PC	1379.91	64.29	230.01	89.44	1763.65
	CH	1387.12	50.72	138.59	212.37	1788.80
average quantity of HS (μg/g)	SM	25.35	47.61	20.34	152.77	246.07
	P	8.22	24.65	40.61	102.71	176.20
	G	9.69	24.26	16.23	-	50.18
	LF	19.29	19.26	18.15	77.29	133.99
	PB	6.14	41.35	18.63	65.12	131.24
	PC	2.25	17.29	11.52	73.28	104.36
	CH	9.50	31.27	9.92	138.07	188.76
average quantity of HA (μg/g)	SM	539.60	38.54	593.58	140.96	1312.68
	P	204.47	15.69	434.21	46.52	700.90
	G	1012.31	199.35	927.24	-	2138.89
	LF	220.28	34.29	34.53	178.82	467.92

	PB	176.80	24.55	308.55	18.21	528.10
	PC	89.70	20.52	106.01	18.73	234.96
	CH	54.27	8.13	10.51	49.01	121.93

Table S3. Percentage of CS disaccharide compositions.

Percentage		CS-0S	CS-2S	CS-4S	CS-6S	SB	SD	SE
Bone	SMB	17.15%	0.14%	30.12%	52.16%	0.15%	0.14%	0.13%
	PB	10.51%	0.21%	46.56%	41.82%	0.22%	0.44%	0.25%
	GB	14.25%	0.53%	29.43%	54.50%	0.83%	0.29%	0.16%
	LFB	10.32%	0.48%	37.17%	50.83%	0.61%	0.34%	0.24%
	PBB	12.95%	0.31%	25.32%	61.20%	0.03%	0.12%	0.07%
	PCB	17.34%	0.18%	27.49%	54.57%	0.03%	0.27%	0.11%
	CHB	12.42%	0.25%	46.70%	39.17%	0.16%	0.61%	0.70%
Muscle	SMM	2.79%	0.15%	85.23%	2.37%	7.95%	0.56%	0.94%
	PM	7.47%	0.27%	67.90%	8.22%	9.95%	0.00%	6.19%
	GM	25.61%	0.09%	56.16%	8.20%	9.07%	0.77%	0.09%
	LFM	11.94%	0.08%	44.97%	37.68%	3.18%	0.48%	1.68%
	PBM	12.41%	0.10%	53.07%	27.64%	5.51%	0.46%	0.81%
	PCM	20.72%	0.44%	61.40%	9.95%	5.26%	0.83%	1.39%
	CHM	18.11%	0.02%	57.25%	16.25%	5.15%	1.27%	1.95%
Skin	SMS	28.09%	0.31%	61.53%	1.87%	7.87%	0.25%	0.07%
	PS	51.48%	0.08%	39.57%	2.83%	5.03%	0.20%	0.81%
	GS	25.61%	0.09%	56.16%	8.20%	9.07%	0.77%	0.09%
	LFS	11.94%	0.08%	44.97%	37.68%	3.18%	0.48%	1.68%
	PBS	12.41%	0.10%	53.07%	27.64%	5.51%	0.46%	0.81%
	PCS	20.72%	0.44%	61.40%	9.95%	5.26%	0.83%	1.39%

	CHS	18.11%	0.02%	57.25%	16.25%	5.15%	1.27%	1.95%
Viscera	SMV	8.43%	0.25%	79.80%	1.60%	5.59%	0.18%	4.15%
	PV	6.58%	0.16%	66.83%	9.11%	9.41%	0.00%	7.90%
	GV	-	-	-	-	-	-	-
	LFV	16.08%	0.33%	57.76%	7.57%	6.60%	0.41%	11.24%
	PBV	13.26%	0.47%	68.95%	7.14%	5.38%	1.09%	3.70%
	PCV	14.64%	0.22%	64.70%	10.47%	5.93%	0.99%	3.04%
	CHY	19.73%	0.16%	58.00%	10.88%	4.68%	0.31%	6.24%

Table S4. Percentage of HS disaccharide compositions.

Percentage		HS-0S	HS-2S	HS-6S	HS-NS	HS-NS6S	HS-NS2S	HS-TriS
Bone	SMB	56.92%	-	3.99%	14.60%	11.29%	11.44%	1.77%
	PB	74.16%	-	2.09%	9.55%	9.50%	4.70%	-
	GB	48.80%	-	3.30%	17.60%	16.44%	12.64%	1.23%
	LFB	47.37%	-	4.77%	19.13%	16.09%	11.26%	1.38%
	PBB	69.46%	-	0.43%	14.32%	6.29%	9.64%	-
	PCB	38.40%	0.18%	5.57%	20.99%	18.41%	16.58%	-
	CHB	71.40%	3.44%	2.69%	10.73%	6.19%	5.56%	-
Muscle	SMM	54.21%	0.63%	6.95%	17.54%	7.64%	10.07%	2.95%
	PM	36.31%	0.28%	6.55%	18.69%	16.70%	17.37%	4.25%
	GM	41.64%	0.08%	7.30%	16.00%	17.06%	12.66%	5.25%
	LFM	36.95%	0.12%	4.75%	17.02%	18.13%	14.66%	8.41%
	PBM	48.82%	0.16%	5.45%	15.89%	10.51%	14.38%	4.86%
	PCM	38.40%	0.18%	5.57%	20.99%	18.41%	16.58%	-
	CHM	40.53%	0.30%	7.62%	18.53%	15.94%	13.59%	3.49%
Skin	SMS	57.92%	0.00%	5.98%	13.48%	12.65%	9.98%	0.00%
	PS	54.88%	0.59%	5.35%	14.21%	13.86%	8.70%	2.71%
	GS	51.67%	-	9.68%	12.15%	15.66%	8.16%	2.67%
	LFS	47.02%	-	5.23%	15.68%	16.58%	8.69%	6.79%
	PBS	53.45%	-	6.42%	15.29%	12.92%	10.05%	1.87%

	PCS	50.27%	-	6.53%	13.10%	17.81%	10.41%	1.87%
	CHS	48.84%	-	5.35%	16.92%	15.53%	12.59%	0.77%
	SMV	51.51%	0.32%	8.30%	13.26%	8.81%	8.46%	9.35%
	PV	39.87%	0.00%	9.28%	17.99%	16.10%	10.10%	6.66%
	GV	-	-	-	-	-	-	-
Viscera	LFV	50.02%	0.08%	6.09%	14.44%	12.21%	7.74%	9.42%
	PBV	49.79%	0.06%	9.61%	14.20%	14.17%	6.45%	5.71%
	PCV	36.19%	0.24%	7.91%	16.21%	16.03%	8.30%	15.12%
	CHY	47.13%	0.27%	7.75%	15.54%	12.09%	9.62%	7.60%

Table S5. Molecular weight of CS from different sources.

Source	Code	Mn (kDa)	Mw (kDa)	PDI
Cartilage (squid)	CSE	139.5	146.40	1.05
Cartilage (shark)	CSC	41.25	56.07	1.23
Trachea (bovine)	CSA	46.72<br>20.17	48.27<br>20.84	1.03<br>1.03
Bone (Gray Plaice)	GB	22.33	24.08	1.08
Bone (*Limanda ferruginea*)	LFB	25.07	27.76	1.11
Bone (*Platichthys bicoloratus*)	PBB	22.67	23.98	1.057
Bone (*Scophthalmus maximus*)	SMB	24.24	25.40	1.05

Table S6. Percentage of CS from the flounder bone disaccharide compositions.

CS Sample	Disaccharide compositions							
	**CS-0S**	**CS-2S**	**CS-4S**	**CS-6S**	**SB**	**SD**	**SE**	**4S/6S**
CSC	1.47%	0.03%	29.37%	61.12%	0.22%	6.88%	0.90%	0.48
SMBCS	10.14%	0.05%	39.22%	48.47%	0.25%	1.71%	0.15%	0.81
PBBCS	6.22%	-	34.23%	57.48%	-	2.07%	-	0.60
LFBCS	8.69%	-	34.64%	54.91%	-	1.77%	-	0.63
GBCS	8.74%	0.03%	23.35%	65.67%	0.06%	2.04%	0.12%	0.36

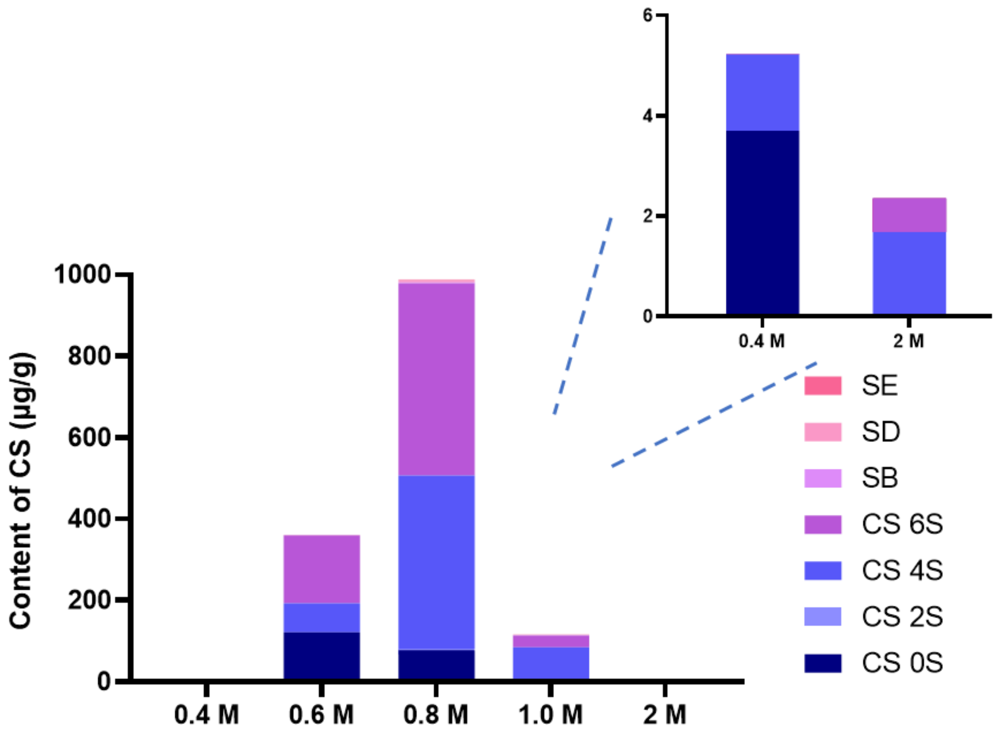

Figure S1. Analysis of CS composition and content of polysaccharide eluting components of SMB.

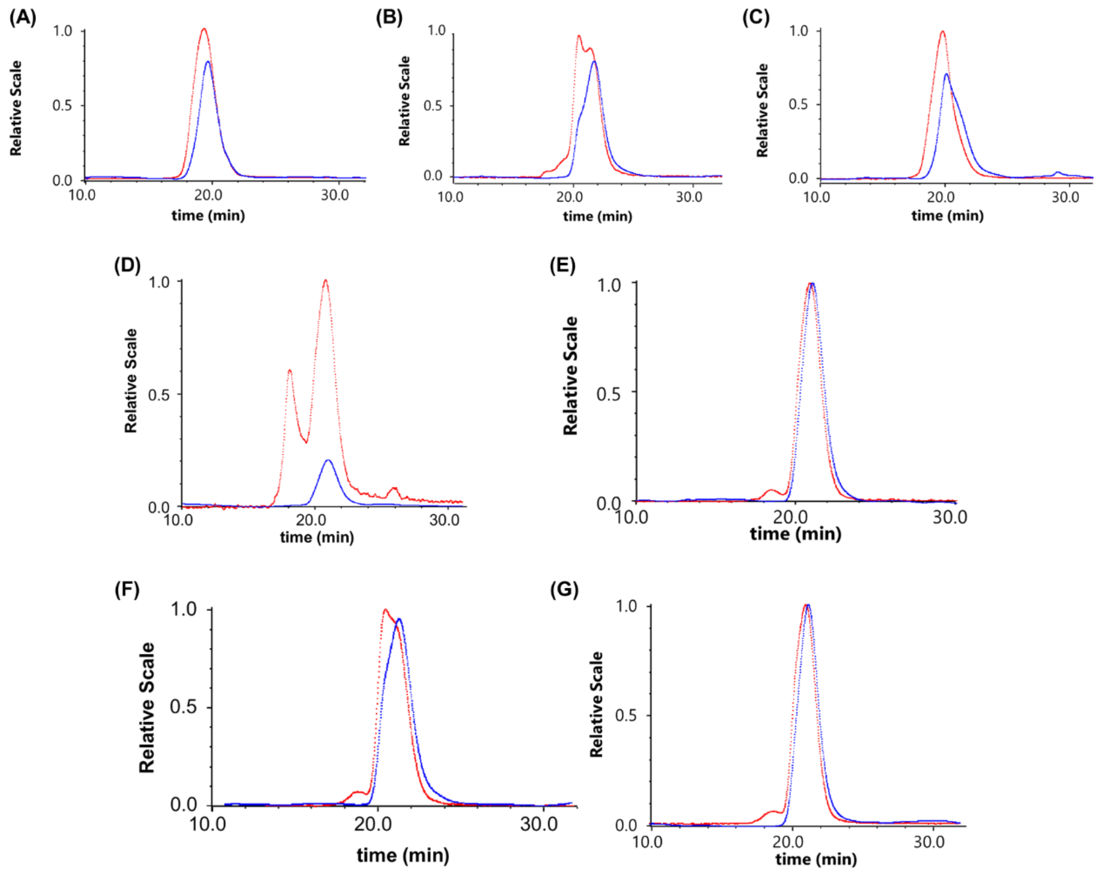

Figure S2. HPGPC-MALLS chromatograms of (A) CSE, (B) CSA, (C) CSC, (D) SMBCS, (E)

PBBCS, (F) LFBCS and (G) GBCS.

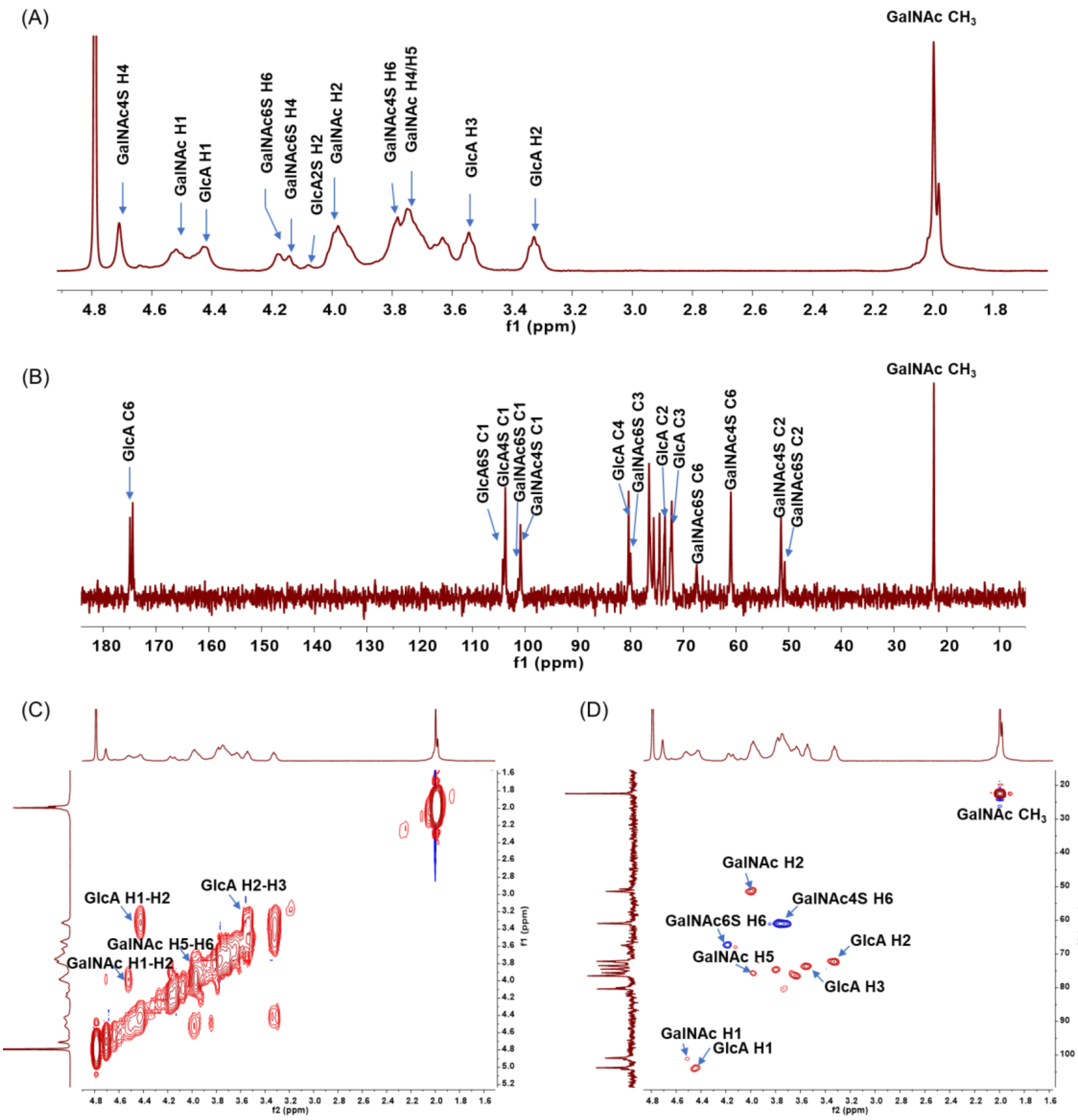

Figure S3. (A) ^{1}H-NMR, (B) ^{13}C-NMR, (C) ^{1}H-^{1}H COSY and (D) ^{1}H-^{13}C HSQC spectroscopy of CSA.

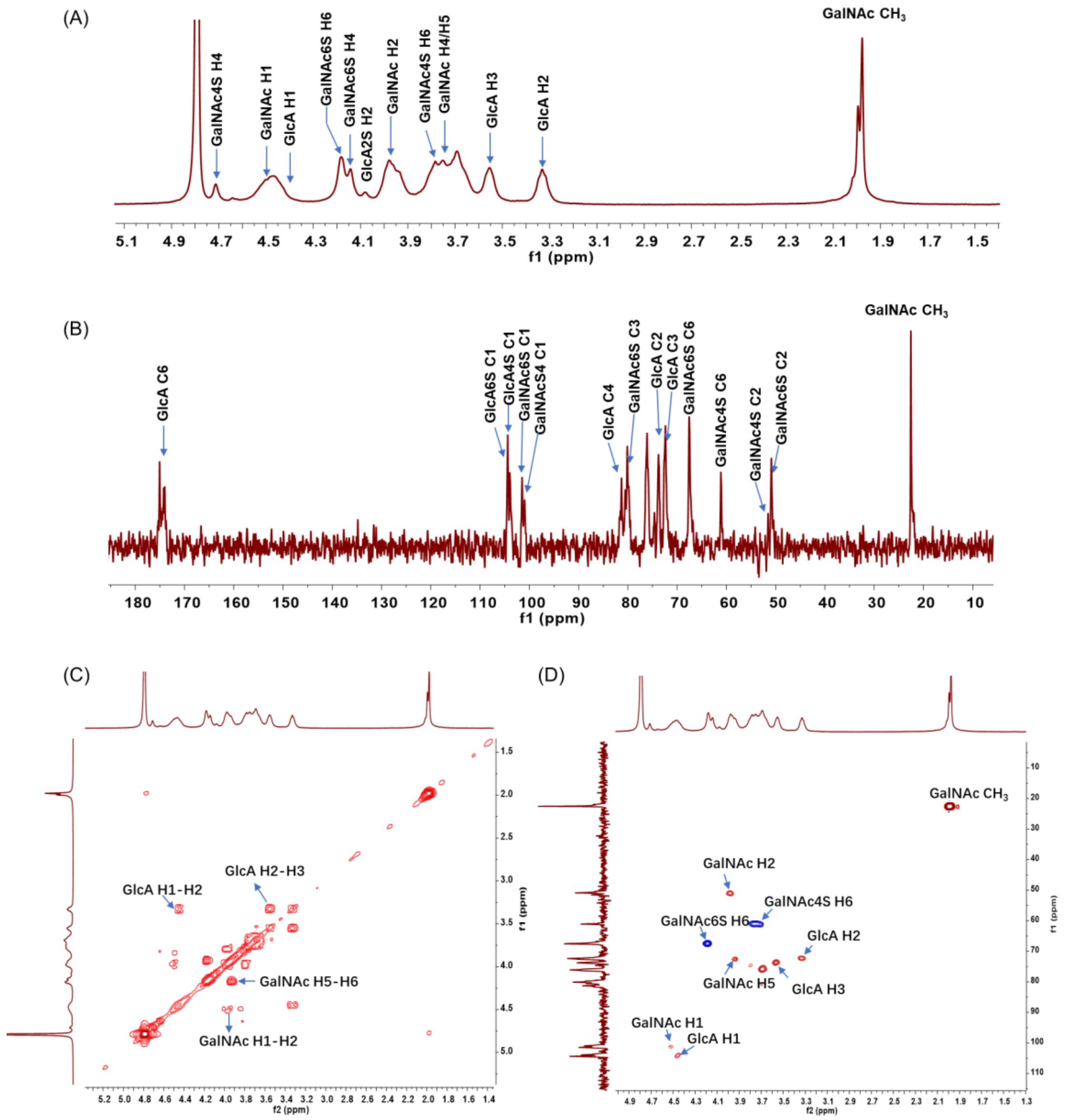

Figure S4. (A) ^{1}H-NMR, (B) ^{13}C-NMR, (C) ^{1}H-^{1}H COSY and (D) ^{1}H-^{13}C HSQC spectroscopy of LFBCS.
